# Supplementary material for: N,N-Dimethylglycine Sodium Salt Exerts Marked Anti-Inflammatory Effects in Various Dermatitis Models and Activates Human Epidermal Keratinocytes by Increasing Proliferation, Migration, and Growth Factor Release
Source: Int J Mol Sci. 2023 Jul 9;24(14):11264. doi: 10.3390/ijms241411264 (PMC10379135; doi:10.3390/ijms241411264)
Supplement: Supplementary file 1 [file ijms-24-11264-s001.zip › ijms-2404209-supplementary.pdf]

Article

N,N-Dimethylglycine Sodium Salt Exerts Marked Anti-Inflammatory Effects in Various Dermatitis Models and Activates Human Epidermal Keratinocytes by Increasing Proliferation, Migration and Growth Factor Release

zzAlexandra Lendvai [1,2], Gabriella Béke [1], Erika Hollósi [1], Maike Becker [3], Jörn Michael Völker [3], Erik Schulze zur Wiesche [3,4], Attila Bácsi [1,5], Tamás Bíró [1] and Johanna Mihály [1*]

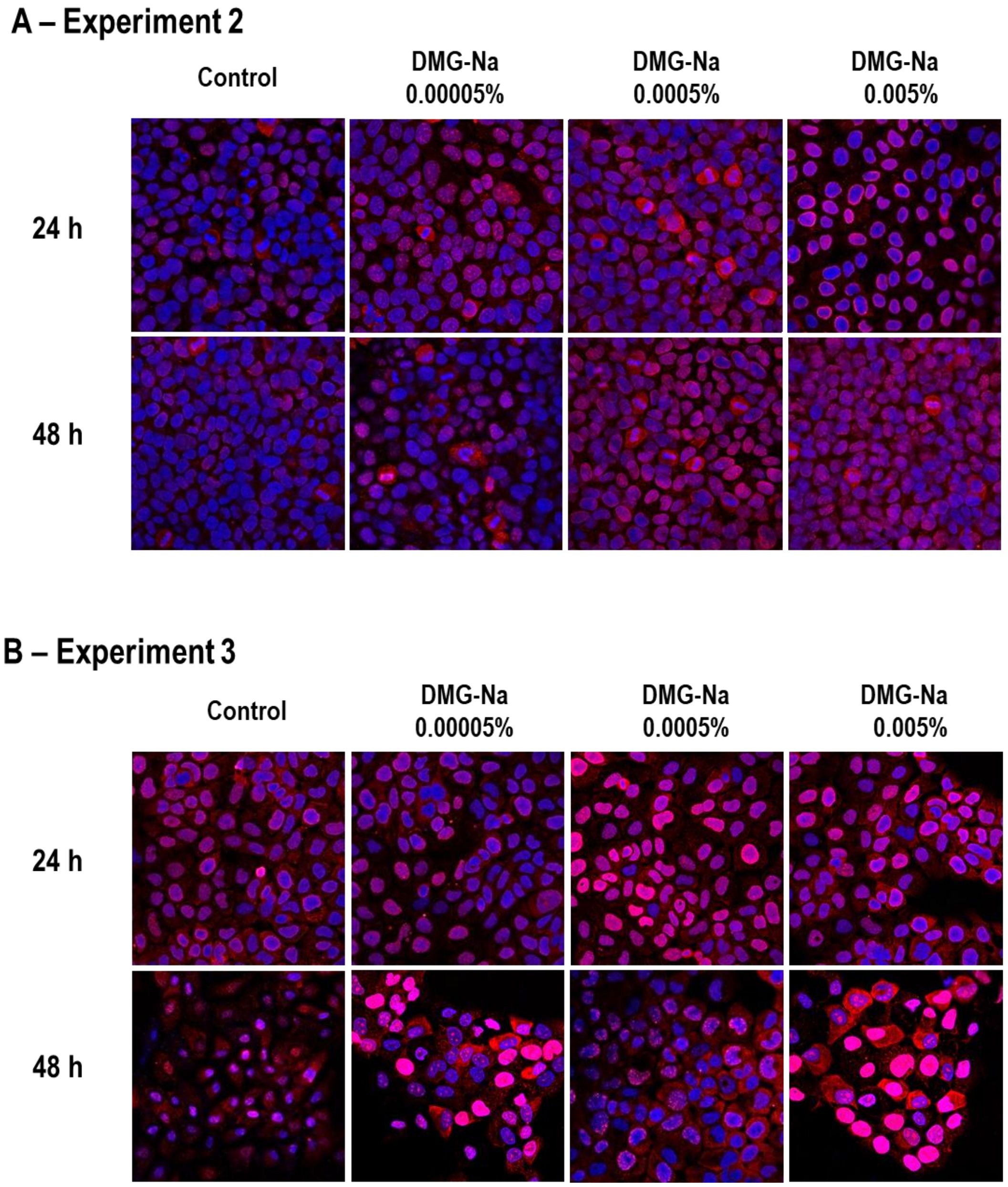

Supplementary Figure S1. Effects of DMG-Na on cellular proliferation of human epidermal HaCaT keratinocytes – Ki67 immunocytochemistry. Effects of various concentrations of DMG-Na on the protein level expression of the proliferation marker Ki67 after 24 and 48 h treatment. **A, B)** Representative images of Ki67 immunofluorescence labeling (Ki67: purple, DAPI: blue) of Experiment 2 and 3 (Experiment 1 is presented in **Figure 1C**).

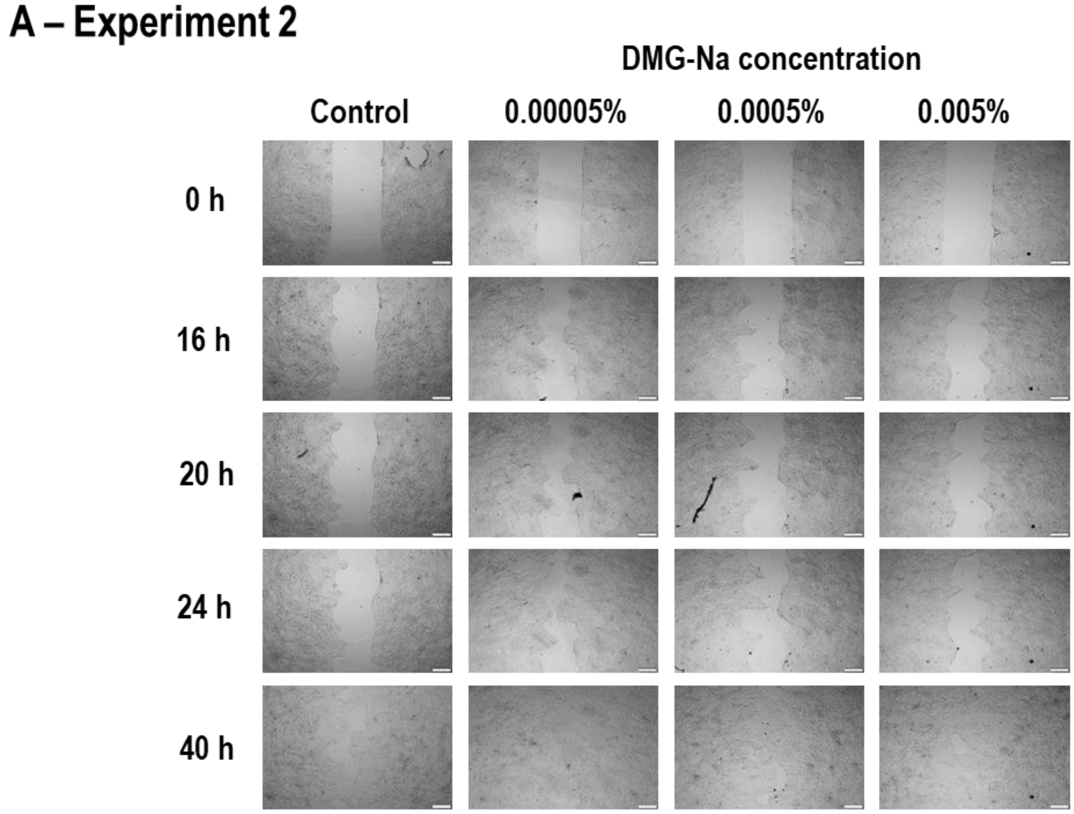

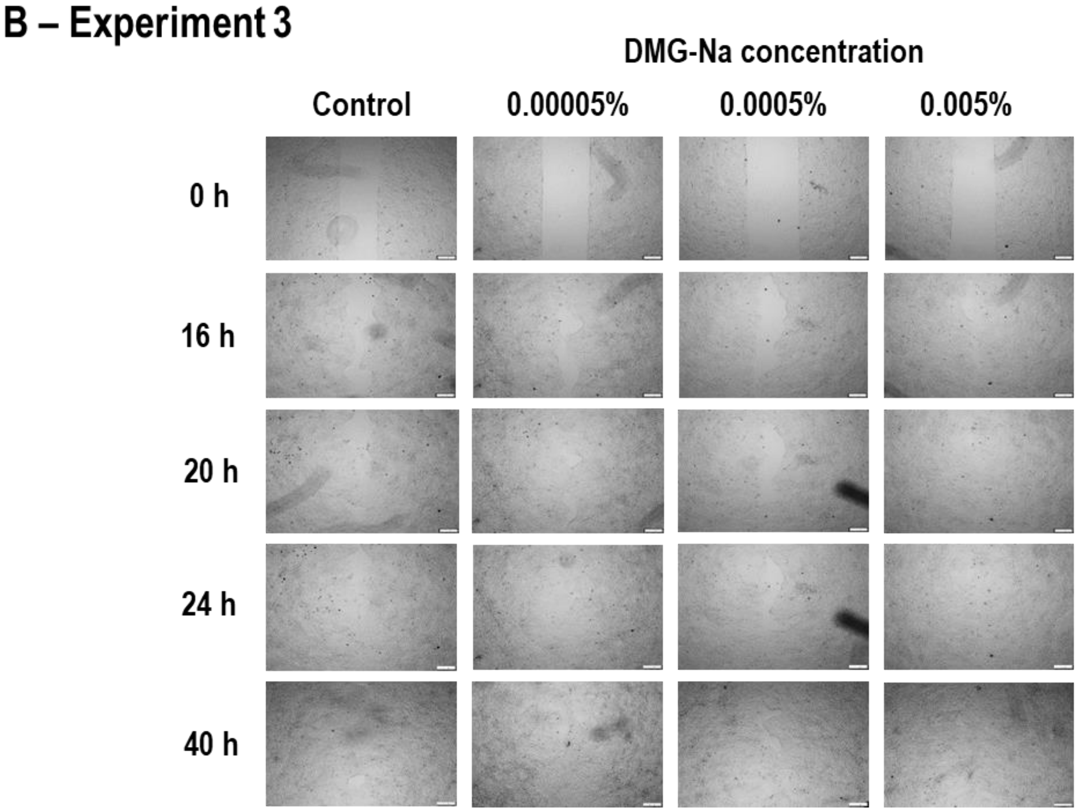

Supplementary Figure S2. Effects of DMG-Na on scratch wound closure (i.e. migration) of human epidermal HaCaT keratinocytes – Representative photomicrographs. Effects of various concentrations of DMG-Na on the migration of HaCaT keratinocytes were assessed at different time-points. Representative photomicrographs of Experiment 2 and 3 (Experiment 1 is presented in **Figure 2A**).

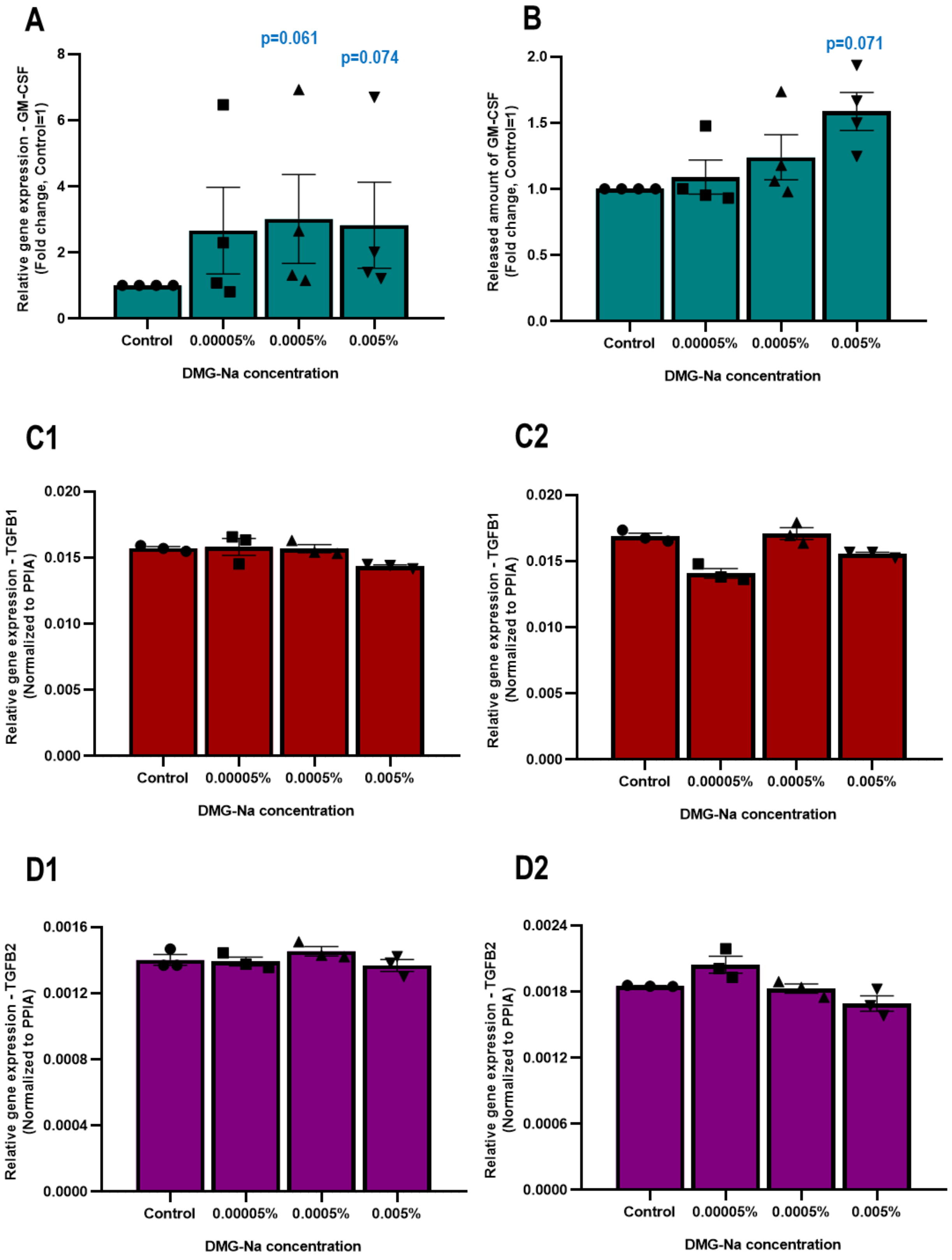

Supplementary Figure S3. Effects of DMG-Na on expression of growth factors in human epidermal HaCaT keratinocytes. **A, B)** Effects of various concentrations (0.00005%, 0.0005% and 0.005%) of DMG-Na on the mRNA level (**A**, qRT-PCR assay) and protein level release (**B**, ELISA assay) of granulocyte-macrophage colony-stimulating factor (GM-CSF) of HaCaT keratinocytes were assessed after 24 h of qRT-PCR and 48 h of ELISA treatment. Both panels show pooled data of independent experiments (n=4). Mean ± SEM. Number shown statistical difference compared to control by ANOVA. **C, D)** Effects of various concentrations (0.00005%, 0.0005% and 0.005%) of DMG-Na on the mRNA level (qRT-PCR assay) of transforming growth factor (TGF)β1 (**C1, C2**) and TGFβ2 (**D1, D2**) of HaCaT keratinocytes were assessed after 24 h in 2 independent experiment. Mean ± SEM.

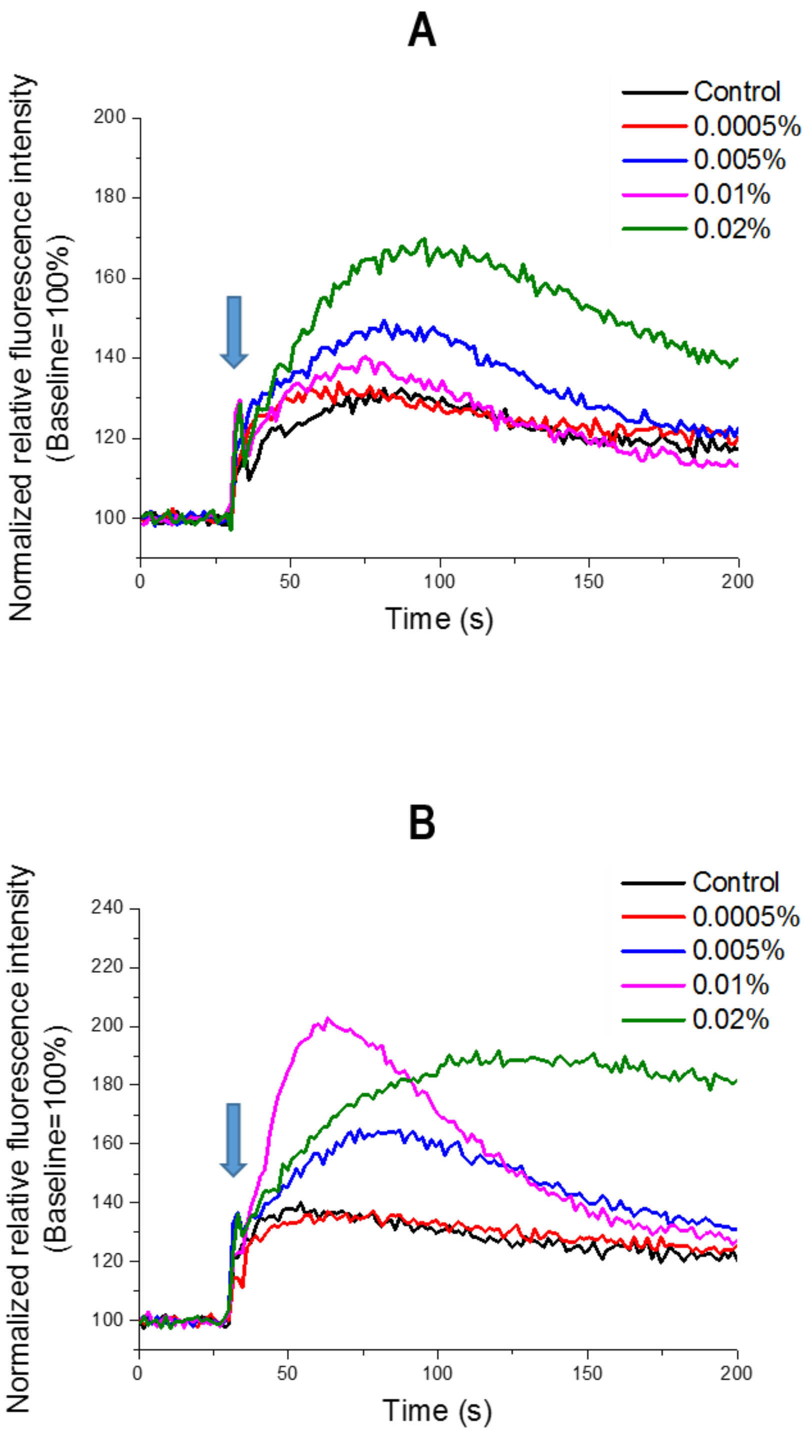

Supplementary Figure S4. Effects of DMG-Na on intracellular Ca concentration ($[Ca^{2+}]_i$) of human epidermal HaCaT keratinocytes. Representative fluorimetric Ca imaging data recorded on Fluo-4–AM-loaded human epidermal HaCaT keratinocytes in 2 independent experiments (**A**, **B**). The arrow indicates the application of various concentrations (0.0005%, 0.005%, 0.01% and 0.02%) of DMG-Na in 1.8 mM Ca^{2+} containing solution.
